# Impact of the ABO and Rh Blood Groups on Malaria Incidence, Recurrence, and Severity Among a Moroccan Military Contingent in the Central African Republic

**DOI:** 10.7759/cureus.88100

**Published:** 2025-07-16

**Authors:** Walid El Faria, Youssef Loubadi, Yasmine Moutmir, Mounir Ababou, Adnane Hammani, El Mehdi Mahtat, Selim Jennane, Hicham El Maaroufi, Kamal Doghmi

**Affiliations:** 1 Hematology, Mohammed V Military Instruction Hospital, Rabat, MAR; 2 Biology and Medical Analysis, Mohammed V Military Instruction Hospital, Rabat, MAR

**Keywords:** abo blood groups, blood type, chemoprophylaxis, falciparum, malaria, plasmodium, plasmodium falciparum, rhesus type

## Abstract

Malaria is a persistent and serious threat to military personnel deployed in endemic regions like the Central African Republic (CAR). While we know our blood type can sometimes influence how our bodies react to malaria, the full picture isn't always clear. We conducted an eight-month study (October 2024 - May 2025) with 749 Moroccan soldiers to investigate if their ABO and Rh blood groups played a role in their risk of contracting malaria, how often they got it, or if they developed severe forms.

It's important to know that these soldiers were diligently following a weekly mefloquine chemoprophylaxis regimen and consistently using physical protective measures like insect repellents and bed nets. During the study, 156 soldiers (20.8%) experienced at least one malaria episode, with a striking 89% of these cases caused by *Plasmodium falciparum*, the most dangerous species. Only eight soldiers (1.1% overall) developed severe malaria.

Despite *P. falciparum'*s strong presence (where blood group O is often reported to offer protection), our statistical analysis found no significant association between a soldier's ABO or Rh blood group and the incidence of malaria, its recurrence or disease severity.

These findings are quite insightful. They suggest that, even when facing the predominant and severe *P. falciparum* strain, rigorous malaria prevention strategies used in this military setting, including effective chemoprophylaxis and consistent physical protection, likely overshadow any inherent genetic influences from blood groups. In environments with strong preventive measures, individual blood type appears to play a less dominant role in determining who gets sick or how severely. Our study reinforces the critical importance of robust and consistently applied malaria prevention programs for protecting military personnel in high-risk zones.

## Introduction

Malaria, caused by Plasmodium parasites and transmitted by the Anopheles mosquito, remains a serious global health crisis, particularly in sub-Saharan Africa. In 2022, the World Health Organization (WHO) estimated that there were 249 million malaria cases and there were 608,000 malaria deaths worldwide, with the large majority occurring in the WHO African Region [1]. If untreated, malaria can progress quickly from mild, uncomplicated forms to severe, critical, or even life-threatening forms, such as cerebral malaria, severe anemia, or acute kidney injury [2].

For the military forces deployed in endemic regions like the Central African Republic (CAR), malaria can impact operational readiness and cause substantial morbidity and mortality [3,4]. Individual susceptibility to malaria, a complex interaction of the host and parasite, can be attributed to several factors. Host genetic factors, including ABO and Rh blood groups, are theorized to negatively affect the susceptibility and severity of malaria illness [4]. Blood group O may represent a protective phenotype against severe *Plasmodium falciparum* malaria, owing to individuals with blood group O having lower rates of rosetting and cytoadherence in vitro [5,6], while blood groups A, B, and AB may exhibit increased susceptibility. However, the evidence is considered inconclusive [7,8]. The Rh factor's role is still unknown [9]. In light of these inconsistencies, this study aims to investigate how the ABO and Rh blood group phenotypes might influence the incidence of malaria, malaria recurrence, and severity of malaria illness in a group of Moroccan military personnel deployed in the CAR, thereby offering insights into the natural history.

## Materials and methods

Study population and data collection

We conducted a retrospective study over eight months, from October 1, 2024, to May 31, 2025. Our study population consisted of Moroccan military personnel deployed in the Central African Republic (CAR) as part of the United Nations Multidimensional Integrated Stabilization Mission in the Central African Republic (MINUSCA). All contingent members were included, and the collected data included each soldier's ABO and Rh blood group, the occurrence of malaria episodes, the number of recurrent malaria episodes, and the severity of each episode.

It's important to note that all military personnel were on malaria chemoprophylaxis with mefloquine 250 mg/week. The tablets were dispensed weekly and their administration was strictly supervised. They also received regular education on the importance of consistent adherence to this prophylaxis and the implementation of physical protective measures. These measures included the use of insect repellent gels, bed nets, and wearing long-sleeved clothing during periods of active biting by the anopheles mosquitoes. 

Malaria diagnosis and severity criteria

Malaria diagnosis followed the contingent's established management protocol. In suspected cases presenting with clinical symptoms of malaria (fever, chills, asthenia, headaches, myalgias, nausea, vomiting, etc.), microscopic diagnosis using Giemsa-stained thick and thin blood smears was performed when the patient was in proximity to the contingent's hospital. For patients deployed in remote areas, diagnosis was conducted via a rapid diagnostic test (RDT) for malaria. A malaria episode was classified as de novo when the patient had no prior history of malaria. Recurrence was defined as the re-emergence of a malarial attack following successful initial treatment or after an asymptomatic period.

The criteria for defining severe malaria episodes were based on the clinical and biological guidelines of the WHO, as detailed in Table 1.

**Table 1:** Severe malaria criteria SpO2: Peripheral arterial oxygen saturation; RR: Respiratory rate.

Clinical criteria	Biological criteria
Neurological impairment	Acidosis (bicarbonates under 15 mmol/L or pH under 7)
Respiratory impairment (SpO2 under 92%, RR over 30)	Hyperlactatemia over 2 mmol/L
Cardiovascular impairment (Signs of shock)	Parasitemia over 4%
Clinical hemorrhage	Renal impairment (creatinine over 30 mg/L, uremia over 20 mmol/µL)
Clinical jaundice associated with hyperparasitemia	Hypoglycemia under 2.2 mmol/L
	Severe anemia associated to hyperparasitemia (Hb under 7 g/dL)

Statistical analysis

Data analysis was performed using Jamovi software (version 2.3.2.4, The Jamovi project, Sydney, Australia). Descriptive statistics were employed to characterize the study population and blood group distribution, with results expressed as percentages. To assess the impact of blood groups on malaria incidence and severity, Chi-squared tests and binary logistic regressions were used. Odds ratios (ORs) with their 95% confidence intervals (95% CI) were calculated. For the analysis of the recurrence of malarial episodes, Welch's ANOVA test (after confirming variance homogeneity using Levene's test) and Tukey's post-hoc tests were applied for mean comparisons. Statistical significance was set at a p-value less than 0.05.

This study was approved by the Ethics Committee of Mohammed V Military Teaching Hospital following the meeting held on May 27, 2025, under reference number CER-17-25/HMIMV. 

## Results

Characteristics of the study population

Our study included a total of 749 Moroccan military personnel. Among them, seven (0.9%) were female soldiers, and the average age was 31 years. During the eight-month observation period, from October 2024 to May 2025, 156 military personnel (20.8%) experienced at least one malarial episode, 44 individuals experienced more than one episode and eight presented with severe malaria (1.1% of the entire cohort and approximately 5.1% of the 156 malaria cases). Concerning Plasmodium species, microscopic diagnosis revealed that 89% of cases were due to *Plasmodium falciparum*, 8% involved *Plasmodium malariae* in co-infection with *P. falciparum*, and 3% were attributed to *Plasmodium ovale*.

The distribution of ABO blood groups within this cohort is shown in Table 2.

**Table 2:** Blood group distribution of study participants (n=749)

Blood group system	Blood group	Percentage (%)
ABO	O	51.8
	A	30.0
	B	14.7
	AB	3.5
Rh afctor	Rh+	90.8
	Rh-	9.2
Combined ABO-Rh	O+	46.6
	A+	27.4
	B+	13.6
	O-	5.2
	AB+	3.2
	A-	2.7
	B-	1.1
	AB-	0.3

Impact of the blood groups

Incidence of Malaria

There appeared to be no significant connection between an individual's ABO blood group or Rhesus factor and their susceptibility to malaria. Statistical tests showed that any observed differences were likely due to chance, and the strength of the relationship, if any, was extremely weak. These observations were confirmed by the results of the binary logistic regression including both ABO and blood groups as predictors of malaria status (Table 3).

**Table 3:** Association between blood groups and malaria incidence

Blood group comparison (Reference: Group O / Rh+)	Odds Ratio (OR)	95% Confidence Interval (CI)	p-value
Group A vs. Group O	1.164	0.7-1.7	0.45
Group AB vs. Group O	1.463	0.5-3.6	0.40
Group B vs. Group O	0.882	0.5-1.5	0.65
Rh-Negative vs. Rh-Positive	0.900	0.5-1.7	0.90

Recurrence of Malarial Episodes

Descriptive analysis showed that among the 156 patients who experienced at least one malarial episode, multiple episodes occurred in 12.8% of those with Group O, 10.3% with Group A, 4.5% with Group B, and 0.6% with Group AB. Regarding the Rh factor, 25.6% of patients with multiple episodes were Rh-positive, compared to 2.6% Rh-negative individuals. However, detailed statistical analysis of ABO and Rh blood groups did not reveal statistically significant differences in the number of recurrent malarial episodes based on blood group (Table 4).

**Table 4:** Association between blood group and frequency of malarial episodes

Blood group system	Test for variance homogeneity	p-value (Levene's Test)	Main comparison test	p-value (Main test)	p-value (Post-hoc)
ABO	Levene's Test	0.275	Welch's Test	0.820	Not significant
Rhesus (Rh)	Levene's Test	0.746	Welch's Test	0.917	0.909

These results suggest that, in our population, ABO or Rh blood groups do not appear to influence the frequency of malaria episodes among the affected military personnel.

Severity of Malarial Disease

The analysis of the impact of blood groups on the incidence of severe malaria was conducted on the 156 military personnel who had suffered at least one malarial episode. Among the eight severe malaria cases, the distribution by blood group was as follows: 2.6% with Group A (four cases out of all Group A personnel in the contingent) developed severe malaria, compared to 1.9% with Group O (two cases), 0.6% with Group B (one case), and no case with Group AB. Regarding the Rh factor, 0.6% of Rh- individuals (one case) developed severe malaria, compared to 4.5% of Rh+ individuals (seven cases). For the combined ABO-Rh groups: 2.6% of severe cases were A Rh+ (four individuals), 0.6% were O Rh- (one individual), 1.3% were O+ (two individuals), and 0.6% were B+ (one individual). No cases were observed in individuals with other blood groups (AB Rh+, A Rh-, B Rh-, AB Rh-).

The limited number of patients with severe malaria precludes drawing a strong conclusion about the association between blood groups and severe malaria. Our statistical analysis did not reveal a significant relationship as detailed in Table 5.

**Table 5:** Association between the blood groups and severe malaria

Test	Variable compared	p-value	Effect size (Cramer's V)	Odds Ratio (95% CI)
Chi-squared test	ABO blood group	0.702	0.09	-
	Rh factor	0.720	0.02	-
	Combined ABO-Rh	0.500	0.183	-
Binary logistic regression	Group A vs. Group O	0.300	-	2.1 (0.44-9.8)
	Group AB vs. Group O	0.990	-	0.99 (0-∞)
	Group B vs. Group O	0.840	-	1.25 (0.12-13)
	Rh-Negative vs. Rh-Positive	0.700	-	1.34 (0.14-12)

These results indicate that, in our cohort, ABO or Rh blood groups were not statistically significant predictors of severe malaria incidence.

## Discussion

Knowing that malaria has been eradicated in Morocco since May 2010 [10], our study provides insight into the risk of malaria occurrence among international travelers in tropical countries (20.8% in our study). This information is rarely found in the literature. This study will allow for a better assessment of risk and emphasizes the importance of preventive measures (chemoprophylaxis and physical measures).

Our study, conducted among a Moroccan military contingent deployed in the CAR, aimed to evaluate the influence of ABO and Rh blood groups on various aspects of malaria: disease incidence, recurrence of episodes, and severity. Unexpectedly, and contrary to some well-established hypotheses in the scientific literature, our results did not show a statistically significant association between ABO or Rh blood groups and any of the malaria outcomes studied.

The absence of the traditionally reported protective effect of blood group O, particularly against severe *P. falciparum* malaria, is a key observation in our study. Blood group O is often associated with increased resistance because O erythrocytes do not express A or B antigens, which *P. falciparum* can utilize for rosetting or adherence to endothelial cells, key mechanisms in severe malaria pathogenesis [6,11]. Several factors may potentially explain this divergence from observations in other studies. The fact that 89% of our malaria cases were due to *P. falciparum* makes the absence of this association particularly intriguing. This is because protection by blood group O is most extensively documented against this specific species [7]. This suggests that other factors might have masked this expected effect in our population.

A major explanatory factor lies in the strict malaria prevention protocols implemented within the military contingent. Soldiers were on chemoprophylaxis with mefloquine 250 mg/week, a recognized and effective antimalarial [12]. Furthermore, they received regular education on the importance of consistent adherence to this prophylaxis and the diligent application of physical protective measures. These included insect repellent gels, bed nets, and wearing long-sleeved clothing during periods of active Anopheles mosquito biting. The combined effectiveness of this mass chemoprophylaxis and physical protection measures likely created a "ceiling effect" or a sufficiently low "background noise," which could have attenuated or masked the intrinsic influence of blood groups on individual susceptibility. In an environment where infectious pressure is significantly reduced by such effective external interventions, genetic factors with small-to-moderate effects might become undetectable [13]. In addition to that, within a military contingent, living conditions and vector exposure are generally more uniform than in a civilian population. This homogeneity of exposure, coupled with preventive measures, could limit the variability needed to reveal susceptibility differences linked to blood groups.

Although our overall sample size was moderate (749 individuals), the very low number of severe malaria cases (only eight cases, approximately 5.1% of infections) significantly limited the statistical power of our analyses to detect associations, even if they were real and of small magnitude. The wide confidence intervals for the ORs in the severity analysis directly reflect this statistical uncertainty. The very low Cramer's V values further confirm that any potential association would be of very small magnitude and difficult to detect with our sample size.

Other individual or environmental factors not captured in our study, such as actual individual adherence to chemoprophylaxis (despite sensitization efforts), genetic variations beyond blood groups (e.g., Glucose-6-phosphate dehydrogenase (G6PD) deficiency or sickle cell traits, which also offer protection against severe malaria [14]), or the precise intensity of local transmission in specific deployment micro-environments, might have a predominant impact on malaria incidence, recurrence, or severity.

The trends observed in our descriptive statistics for severity (e.g., a slightly higher percentage of severe malaria in Group A and Rh+ individuals compared to Group O and Rh- individuals) were not statistically significant. However, these trends warrant further exploration in studies with increased statistical power.

Our study has several limitations. It is an observational study, which limits our ability to establish direct causal links. The absence of data on Plasmodium species for every infection (though *P. falciparum* predominated overall) and parasite load might have limited the granularity of our severity analysis. While prevention protocols were in place, individual variations in actual adherence to these measures or other behavioral risk factors could not be fully controlled. Finally, the population is highly homogeneous (young military personnel), which may restrict the generalizability of our findings to civilian populations.

Despite the non-significant findings, our results are important as they contribute to the literature on malaria in military settings. They suggest that in populations subjected to rigorous prevention measures and effective chemoprophylaxis, the impact of genetic factors like blood groups might be overshadowed by the effectiveness of these interventions. Our study can serve as a baseline for future research investigating the impact of blood groups, while also considering other potential factors, particularly genetic factors (e.g., G6PD deficiency, sickle cell anemia, etc.).

## Conclusions

In conclusion, our study among a Moroccan military contingent deployed in the CAR did not demonstrate a statistically significant association between ABO or Rh blood groups and the incidence, recurrence, or severity of malaria. These non-significant findings are nevertheless crucial. They suggest that in the context of a military deployment where rigorous prevention protocols, including mefloquine chemoprophylaxis and well-applied physical measures, are implemented, the influence of blood groups on individual malaria susceptibility might be masked or less predominant than collective and individual prevention efforts. This reinforces the continuous and primary importance of malaria prevention and control strategies in military settings for protecting the health of armed forces.
